# Nucleic acid amplification strategies for volume-amplified magnetic nanoparticle detection assay

**DOI:** 10.3389/fbioe.2022.939807

**Published:** 2022-08-10

**Authors:** Zhongchao Huang, Jing Li, Hongwen Zhong, Bo Tian

**Affiliations:** Department of Biomedical Engineering, School of Basic Medical Science, Central South University, Changsha, Hunan, China

**Keywords:** magnetic biosensing, molecular amplification, magnetic nanoparticles, Brownian relaxation, homogeneous assays, volumetric sensing

## Abstract

Magnetic nanoparticles (MNPs) can be quantified based on their magnetic relaxation properties by volumetric magnetic biosensing strategies, for example, alternating current susceptometry. Volume-amplified magnetic nanoparticle detection assays (VAMNDAs) employ analyte-initiated nucleic acid amplification (NAA) reactions to increase the hydrodynamic size of MNP labels for magnetic sensing, achieving attomolar to picomolar detection limits. VAMNDAs offer rapid and user-friendly analysis of nucleic acid targets but present inherence defects determined by the chosen amplification reactions and sensing principles. In this mini-review, we summarize more than 30 VAMNDA publications and classify their detection models for NAA-induced MNP size increases, highlighting the performances of different linear, cascade, and exponential NAA strategies. For some NAA strategies that have not yet been reported in VAMNDA, we predicted their performances based on the reaction kinetics and feasible detection models. Finally, challenges and perspectives are given, which may hopefully inspire and guide future VAMNDA studies.

## Introduction

Magnetic micron/nano-sized particles stand out in comparison to alternative nanomaterials due to their unique properties, for example, easy manipulation, biocompatibility, signal stability, and high contrast (against the inherently negligible magnetic susceptibilities of biological objects), allowing for extensive applications in biomedical engineering (Gloag et al., 2019; Moerland et al., 2019; Cheng et al., 2021). Magnetic nanoparticles (MNPs), including micron-sized particles consisting of nanocomposites, can be directly analyzed as labels and transducers by magnetic sensors, facilitating an ideal mix-and-read biosensing approach that is attractive for on-site analysis and point-of-care testing (Schrittwieser et al., 2016; Xianyu et al., 2018). Based on the sensing principle, MNP detection sensors can be classified as surface-based and volumetric sensors (Issadore et al., 2014). Surface-based MNP sensing (e.g., micro-Hall sensing and giant magnetoresistance sensing) detects the MNP’s magnetic stray field induced by an external magnetic field. Due to the fast decay of the magnetic stray field with distance, only MNPs located close to the surface of the sensing element can be detected with the requirement of separation steps to remove unbound MNPs. In contrast, volumetric magnetic sensing could measure the presence and/or the property (e.g., relaxivity) changes of MNPs dispersed in the entire suspension, which is simple and convenient (Lee et al., 2015), especially when combined with homogeneous reaction strategies. The hydrodynamic size changes of MNPs are related to several magnetic properties such as Brownian relaxation frequency and magnetic anisotropy, allowing the volume-amplified magnetic nanoparticle detection assay (VAMNDA) (Strömberg et al., 2008) based on different volumetric magnetic sensors.

**FIGURE 1:**
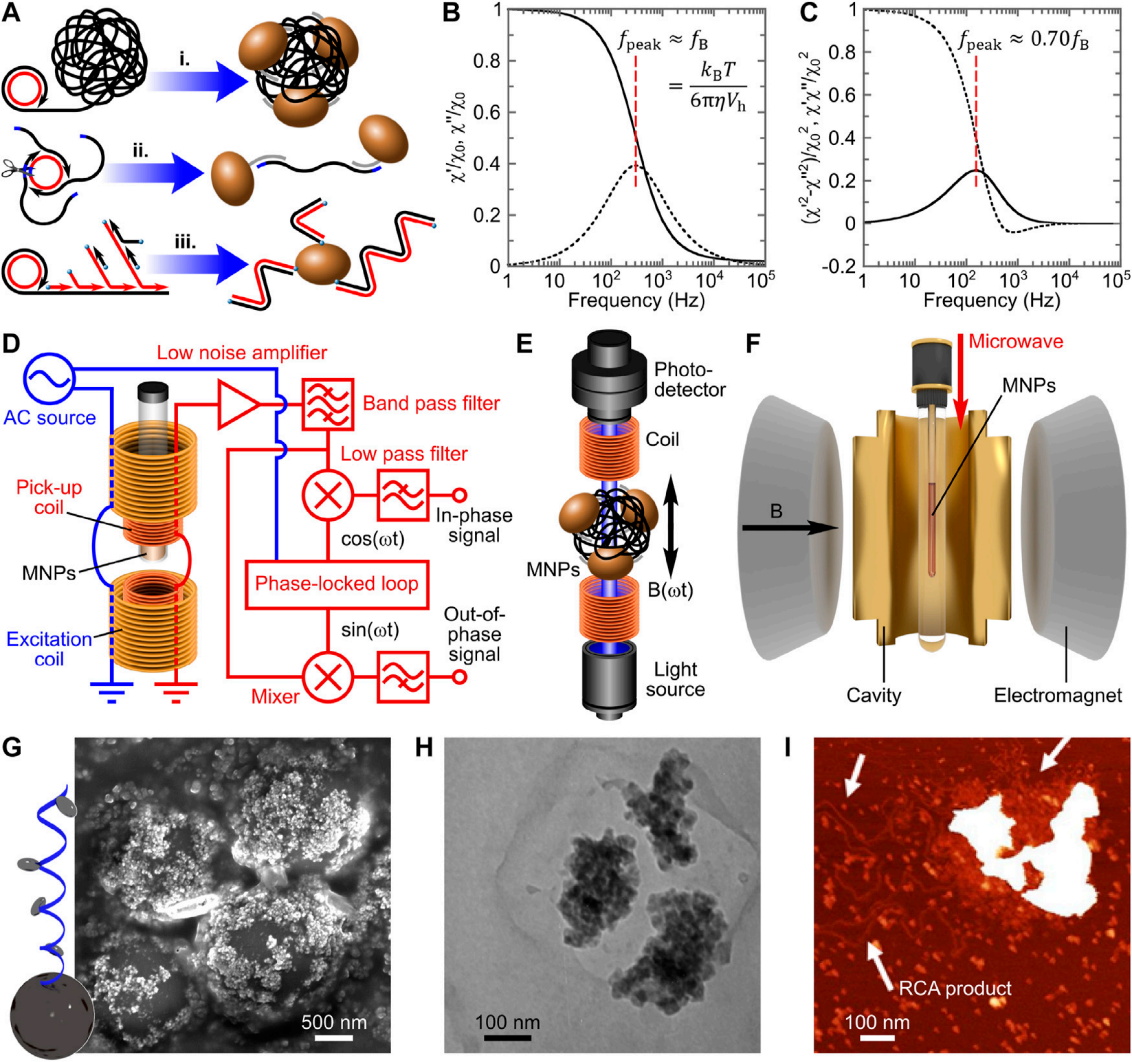
The basic principle of volume-amplified magnetic nanoparticle detection assays. **(A)** Three typical detection models for the nucleic acid amplification-based hydrodynamic size increase of MNPs, instanced by conventional, nicking-enhanced, and hyperbranched rolling circle amplification strategies, respectively. **(B)** Spectra of normalized in-phase and out-of-phase magnetic susceptibility ( $\text{χ}\prime /{\text{χ}}_{\text{0}}$ and $\text{χ}\prime \prime /{\text{χ}}_{\text{0}}$ ), represented by solid and dashed lines, respectively. **(C)** Spectra of normalized in-phase and out-of-phase optomagnetic output, corresponding to $\left({\text{χ}}^{\prime 2}-{\text{χ}}^{\prime \prime 2}\right)/{\text{χ}}_{\text{0}}^{2}$ and $\text{χ}\prime \text{χ}\prime \prime /{\text{χ}}_{\text{0}}^{2}$ in AC susceptometry, represented by solid and dashed lines, respectively. Panels **(B,C)** are adapted with permission from Tian et al. (2017), Copyright 2017, American Chemical Society. Detection schematics of **(D)** AC susceptometer (with a lock-in amplifier), **(E)** optomagnetic sensor, and **(F)** ferromagnetic resonance spectrometer. Panel **F** is adapted with permission from Tian et al. (2018a), Copyright 2018, American Chemical Society. **(G)** Scanning electron microscopy micrograph of core-satellite magnetic superstructures with 100 nm MNP satellites. Adapted with permission from Tian et al. (2018b), Copyright 2017, American Chemical Society. **(H)** Transmission electron microscopy micrograph of 130 nm MNPs aggregated by a DNA coil (only a salt precipitate can be observed). Adapted with permission from Akhtar et al. (2010), Copyright 2010, American Chemical Society. **(I)** Atomic force microscopy micrograph of 100 nm MNPs aggregated by a DNA coil. Adapted with permission from Oropesa-Nuñez et al. (2020), Copyright 2020, American Chemical Society.

Various nucleic acid amplification (NAA) strategies have been employed to increase the hydrodynamic volume of MNPs in VAMNDA. The hydrodynamic size increase of MNPs can be induced by analyte-initiated NAA in three detection models: (I) micrometer-sized single-stranded tandem amplicon coil-induced MNP aggregation, (II) amplicon monomer-mediated MNP linkage, and (III) double-stranded tandem amplicon chain-based MNP “hair growth,” as illustrated in Figure 1A i, ii, and iii, respectively. In VAMNDA, the NAA-induced hydrodynamic size changes of MNPs are usually analyzed by alternating current (AC) susceptometry (or equivalent methodologies), measuring the susceptibility of MNPs exposed to an AC magnetic field. For commonly used MNPs with diameters of 30–300 nm, the MNP’s relaxation process after switching off the external field is dominated by thermal rotational diffusion, that is, Brownian relaxation (Strömberg et al., 2007b). In AC susceptometry, for the simplest case, the Brownian relaxation frequency $f_{\text{B}}$ of the MNP is found as the peak position of the out-of-phase magnetic susceptibility (χ″) spectrum shown in Figure 1B (Cole and Cole, 1941; Strömberg et al., 2007a): $f_{\text{peak}}\approx f_{\text{B}}=k_{\text{B}}T{\left(6\text{π}\eta V_{\text{h}}\right)}^{-1}$ , where $k_{\text{B}}T$ is the thermal energy, η is the viscosity of the suspension, and $V_{\text{h}}$ is the hydrodynamic volume of MNP. Accordingly, AC susceptometry can be applied for analyzing the concentration (related to the peak amplitude) and the hydrodynamic size (related to the peak frequency) of MNP objects, enabling the quantification of the target molecule initiating NAA. Most of the other magnetic sensing principles used in VAMNDA, such as optomagnetic sensor (Donolato et al., 2015a; Fock et al., 2017a, 2017b) and anisotropic magnetoresistance sensor (Østerberg et al., 2013a; 2013b, 2014), were designed referring to the AC susceptometry (Figure 1C). Detection schematics of AC susceptometer (with a lock-in amplifier), optomagnetic sensor, and ferromagnetic resonance spectrometer are illustrated in Figures 1D, E, and F, respectively. The critical performances of these VAMNDAs, for example, the limit of detection (LOD) and the total assay time, are determined mainly by the NAA strategy (Xiao et al., 2022). Herein, we introduce the concepts and performances of different NAA strategies employed in VAMNDAs, followed by the discussion of challenges and perspectives in this technique, which may hopefully inspire and guide future VAMNDA studies.

## Rolling circle amplification

Owing to its simplicity, robustness, and high efficiency, rolling circle amplification (RCA) is the most widely adopted isothermal NAA in biomedical engineering (Ali et al., 2014; Soares et al., 2021; Yao et al., 2021). RCA is the most effective linear amplification due to (I) the conformational stresses that facilitate the strand displacement (Joffroy et al., 2018) and (II) the high polymerizing processivity of the polymerase (whereas in linear template-based amplification reactions, polymerases undergo cycles of diffusion-polymerization-dissociation). In phi29 polymerase-based RCA, ca. 10^5^ nt-long single-stranded tandem amplicons (about 10^3^ times amplification) can be synthesized in 1 h (Banér et al., 1998). Moreover, a single-nucleotide specific process, that is, padlock probe ligation, is usually employed to prepare circular templates for the following RCA, offering the whole reaction system an ability of single-nucleotide discrimination (Nilsson et al., 2000). RCA amplicons form micrometer-sized DNA coils in the aqueous solution, which can be quantified by VAMNDA model I (Figure 1A, i). Figure 1G shows a representative scanning electron microscopy micrograph of RCA coil-aggregated MNPs on the surface of microbeads, demonstrating the formation of micrometer-sized MNP aggregates (Tian et al., 2018b). RCA coil-aggregated MNPs have dramatically increased hydrodynamic volumes characterized by a χ″ peak located at low frequencies (usually out of the detection window). As the remaining unbound MNPs instead of the aggregated MNPs are detected, high signal homogeneity can be obtained, allowing multiplex detection employing MNP labels of different sizes (with distinguishable χ″ peaks) (Strömberg et al., 2009, 2014; Tian et al., 2017).

For RCA-based end-point VAMNDA, where an additional hybridization step is required to anneal probe-modified MNPs onto the coils, 1–10 pM, LODs were reported with total assay times around 2 h (including 1 h of RCA) and dynamic detection ranges of approximately two orders of magnitude (Donolato et al., 2015b; Bejhed et al., 2015; Blomgren et al., 2018; Sepehri et al., 2018; Tian et al., 2019b). By using a microfluidic sample handling system with a simultaneous differential sensor, Sepehri et al. improved the picomolar LOD to 45 fM, being the most sensitive conventional RCA-based VAMNDA (Sepehri et al., 2019). However, in the end-point detection format, MNPs can probably be stopped at the surface of RCA coils due to the multivalent binding reaction, resulting in a limited aggregation effect (Zardán Gómez de la Torre et al., 2010). Transmission electron microscopy and atomic force microscopy studies showed that only a few particles were bound to each RCA coil (Figures 1H, I), suggesting potentially higher sensitivity of the real-time MNP aggregation-based assay (Akhtar et al., 2010; Oropesa-Nuñez et al., 2020). In order to increase the MNP-to-coil ratio and shorten the total assay time, real-time VAMNDAs were presented by performing RCA and MNP hybridization simultaneously with magnetic incubation (i.e., applying magnetic actuation to enhance the binding kinetics) and optomagnetic phase lag sensing, which could achieve an LOD of 0.3 pM. within 90 min (Tian et al., 2019a; Minero et al., 2020).

Due to the steric hindrance and electrostatic repulsion, real-time RCA can hardly achieve the theoretical highest sensitivity (Zardán Gómez de la Torre et al., 2010). Nicking-enhanced RCA (NickRCA) performs nicking reactions during RCA, generating single-stranded amplicon monomers instead of amplicon coils (Li et al., 2010). Moreover, NickRCA allows several polymerases to act simultaneously on one single circular template, which further improves the amplification efficiency (Tian et al., 2019a). Amplicon monomers can bridge MNPs for turn-on measurement (detection model II, Figure 1A, ii). By real-time optomagnetic sensing of MNP phase lag in response to an AC field, NickRCA achieved an LOD of 15 fM target DNA with a total assay time of ca. 100 min (Tian et al., 2019a), which is the lowest LOD obtained by linear amplification-based VAMNDAs. As a comparison, optomagnetic analysis of synthetic DNA monomers (without NAA) presented an LOD of 50 pM. (Mezger et al., 2015), suggesting amplification of NickRCA of ca. 3 × 10^3^ times.

Linear RCA-based VAMNDAs have been demonstrated for the quantification of bacterial/viral sequences (representing, e.g., influenza virus), discrimination of drug-resistance single-nucleotide mutations of *Mycobacterium tuberculosis*, and biplex detection of *Vibrio cholerae* and *Escherichia coli*. However, LODs at a 10^−14^ M range for nucleic acid targets are still inferior for many clinical applications (e.g., virus detection), suggesting the utilization of more efficient NAA strategies.

## RCA-based cascade amplification

A cascade amplification strategy consists of tandemly performed amplification reactions, where the product of one amplification reaction is the trigger, primer, or template of the subsequent amplification reaction. Cascade amplification strategies are versatile to involve various tool enzymes at the expense of complicated strategy design (e.g., polymerases with exonucleolytic activity can digest the intermediate amplicons), especially for the one-pot homogeneous cascade amplification strategies that are preferred for VAMNDA. By stepwise performing n round of linear amplification reactions with the same amplification efficiency $E_{\text{li}}$ , a cascade amplification obtains an ideal efficiency of $E_{\text{ca}}={\left(E_{\text{li}}/n\right)}^{n}$ . Therefore, under ideal conditions, the maximum cascade amplification efficiency, $E_{\text{ca},\text{ max}}=e^{E_{\text{li}}/e}$ , can be achieved at $n=E_{\text{li}}/e$ . However, for homogeneous cascade amplification, $E_{\text{hca}}={\left(E_{\text{li}}\right)}^{n}$ . In reality, however, considering the complicated design/operation and efficiency losses, only two to three reactions are tandemly incorporated for target detection.

**FIGURE 2:**
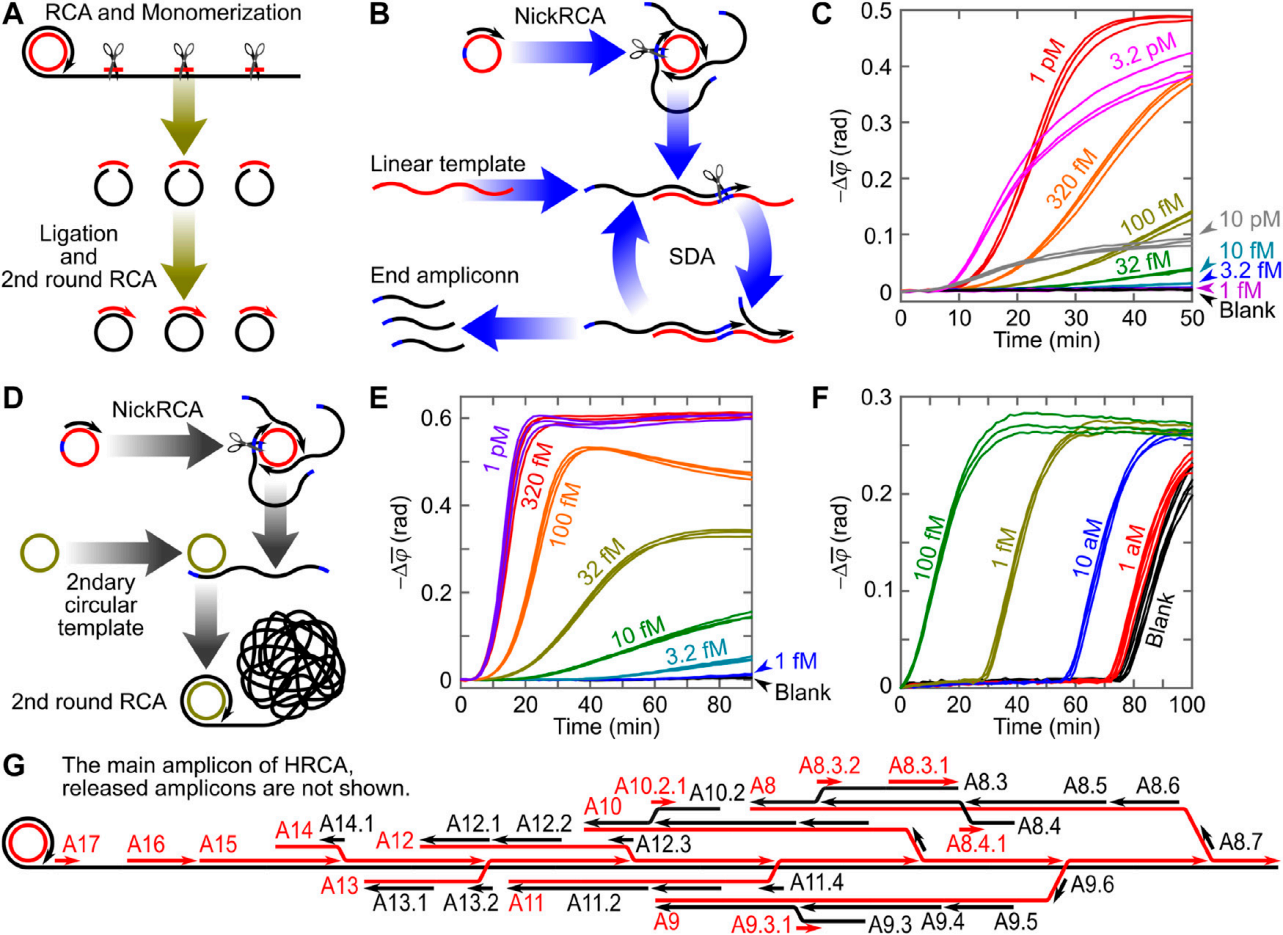
**(A)** Schematic illustration of conventional C2CA. **(B,C)** Schematic illustration and time-resolved optomagnetic signals (phase lag increases) of NECA-based VAMNDA. **(D,E)** Schematic illustration and time-resolved optomagnetic signals of HC2CA-based VAMNDA. **(F)** Time-resolved optomagnetic signals of PG-RCA-based VAMNDA. Panels **(C,E,F)** are adapted with permission from Tian et al. (2020a, 2020b, 2020c), Copyright 2020, Elsevier and Oxford University Press. **(G)** Illustration of HRCA processes on the RCA coil (amplicons that have already been released from the RCA coil are not shown). A8 means the eighth amplicon produced on the RCA coil (A1-A7 have been released), and A8.3 means the third amplicon produced on A8 (A8.1 and A8.2 have been released).

Circle-to-circle amplification (C2CA, illustrated in Figure 2A) is a representative cascade amplification that converts RCA amplicon coils into multiple circular monomers (through endonuclease monomerization followed by ligation) as templates for subsequent RCA (Dahl et al., 2004). The ideal gain of C2CA (i.e., the quantity of generated amplicon) is $A_{\text{C}2\text{CA}}=E_{\text{RCA}}^{n}\prod_{i=1}^{n}t_{i}$ , where $E_{\text{RCA}}$ is the amplification efficiency of RCA and $t_{i}$ is the reaction time of RCA round i . For immuno-recognition of *Bacillus globigii* spores followed by a proximity ligation assay and C2CA for VAMNDA, LODs of ca. 500 and 50 spores were reported by incorporating two and three RCA rounds, respectively (Zardán Gómez de la Torre et al., 2012). Other C2CA-based VAMNDA studies also suggested sub-femtomolar LODs for the bacterial DNA (synthetic and patient samples containing *Escherichia coli* sequences) but without showing a systematic dose-response curve or signal-noise analysis (Göransson et al., 2010; Mezger et al., 2015). Despite the high sensitivity, conventional C2CA requires tedious and time-consuming stepwise operations with different reaction temperatures (since monomerization and ligation are incompatible processes) and can only be analyzed in an end-point format (model I or II).

For homogeneous cascade amplification, nicking-assisted on- and off-loop enzymatic cascade amplification (NECA, illustrated in Figure 2B) comprises a NickRCA to generate primers of a subsequent strand-displacement amplification (SDA) for VAMNDA model II (Tian et al., 2020a). Both NickRCA and SDA were designed with the same template sequence: a padlock probe ligation process was employed to produce the circular template of NickRCA, leaving unligated padlock probe molecules in the suspension served as the template of SDA. Before all the templates are occupied, the ideal gain of NECA follows a quadratic function, $A_{\text{NECA}}=E_{\text{NickRCA}}E_{\text{SDA}}t^{2}$ , where t is the reaction time and $E_{\text{NickRCA}}$ and $E_{\text{SDA}}$ are the efficiencies of NickRCA and SDA, respectively. After all the templates are occupied, amplicons are produced linearly with time, resulting in a quadratic-linear time-resolved signal increase (Figure 2C) with an LOD of 2 fM (detecting a synthetic Dengue virus sequence) obtained in ca. 70 min (Tian et al., 2020a).

Homogeneous C2CA (HC2CA, illustrated in Figure 2D) employs NickRCA to produce primers for the subsequent RCA, combining two amplification reactions in a one-pot homogeneous reaction to eliminate additional monomerization and ligation steps (Tian et al., 2020b). However, the secondary circular template competes with the detection probe. Thus, the RCA coils can hardly be monitored before all circular templates are occupied (either by a primer or by an amplicon coil), indicated by a dead time during which no signal can be detected: $t_{\text{dead}}=\sqrt{2m{\left(E_{\text{NickRCA}}E_{\text{RCA}}x\right)}^{-1}}$ , where m is the concentration of the circular template and x is the target concentration. For $t>t_{\text{dead}}$ , a linearly increased signal can be observed (Figure 2E) with an ideal gain of $A_{\text{HC}2\text{CA}}=\left(t-t_{\text{dead}}\right)\sqrt{2mE_{\text{NickRCA}}E_{\text{RCA}}x}$ . For the quantification of a synthetic SARS-CoV-2 RdRp sequence based on detection model I, HC2CA presented an LOD of 0.4 fM with a total assay time of ca. 100 min (Tian et al., 2020b). Although sensitive and robust (against background amplification), homogeneous cascade amplification strategies such as NECA and HC2CA are difficult to design and optimize, which limits their applications.

## Exponential amplification

An exponential amplification reaction, for example, the polymerase chain reaction (PCR), generates amplicons serving as the trigger, primer, or template of the reaction itself. NickRCA can react exponentially if the circular templates are added as reagents (instead of being prepared by padlock probe ligation), which is known as primer-generation RCA (PG-RCA) (Murakami et al., 2009). PG-RCA amplicons can trigger the formation of MNP dimers for detection model II, obtaining an attomolar LOD for a synthetic Dengue virus sequence (Figure 2F) (Tian et al., 2020c). However, the rapid generation of amplicon monomers requires real-time MNP binding. Otherwise, MNPs could be saturated by an excess amount of amplicons without a significant hydrodynamic size increase. Considering that some exponential NAAs include either high-temperature processes or molecular crowding agents, real-time VAMNDAs based on these NAAs are challenging. This may explain why PCR, EXPAR (exponential amplification reaction), and RPA (recombinase polymerase amplification) have not been reported in VAMNDA so far.

Loop-mediated isothermal amplification (LAMP) produces double-stranded amplicons of different lengths. By using biotinylated primers, LAMP amplicons can attach to the streptavidin-modified MNPs for VAMNDA model III, which can be achieved by either an end-point MNP binding or a real-time on-particle amplification, resulting in attomolar LODs for different synthetic (e.g., a Zika virus sequence) and real (e.g., Newcastle disease virus) targets within 30 min (Tian et al., 2016b, 2016c; Minero et al., 2017). Except for the amplicon-based detection, precipitation of Mg_2_P_2_O_7_ (a by-product of LAMP) onto MNPs can also be utilized for VAMNDA, providing a sub-femtomolar LOD by using a ferromagnetic resonance spectrometer (Tian et al., 2018a). Similar to LAMP, hyperbranched RCA (HRCA) is an exponential amplification strategy producing double-stranded amplicons of different lengths. HRCA employs a pair of primers to trigger SDA on the RCA coil (Figure 2G) with a gain of $A_{\text{HRCA}}\propto 2^{E_{\text{SDA}}t}$ . Although HRCA has not yet been reported in VAMNDA, we predict that HRCA-based VAMNDA can be realized in the detection model III (Figure 1A, iii).

## Challenges and perspectives

Existing VAMNDA models present inherence defects: model I requires long single-stranded amplicons that can hardly be produced with high efficiency; model II has to discriminate MNP dimers from a background of individual MNPs; model III is limited by the length distribution of amplicons and cannot distinguish false-positive amplicons. Moreover, the sensitivity of VAMNDA is determined mainly by the amplification efficiency of the NAA strategy, implying that exponential NAAs are preferred for lower LODs. However, due to the mispriming, *ab initio* DNA synthesis, and polymerase side-reactions, nonspecific synthesis of false-positive products is inevitable in highly effective NAA reactions, especially the exponential ones (Zyrina and Antipova, 2021). A CRISPR/Cas12a-based internal negative control system was reported in combination with VAMNDA, which could indicate the onset of nonspecific amplification (Tian et al., 2020c). Nevertheless, such a warning system cannot suppress nonspecific amplification. We expect that the problem of nonspecific synthesis can hopefully be solved in the future by applying highly accurate and programmable techniques such as DNA logic gates.

For the purpose of system automation and miniaturization, one-pot homogeneous reactions are preferred for VAMNDA. However, clinical applications of NAA strategies usually consist of a series of processes such as analyte extraction and purification. The lab-on-a-disc technique can integrate multi-step assays into a chip on which density gradient centrifugation and centrifugo-pneumatic valving can be processed, facilitating a compact and fully automated sample-to-answer biosensor (Uddin et al., 2018). In another RCA-based VAMNDA study (Garbarino et al., 2019), all the detection processes, including target capture, padlock probe ligation, molecular amplification, and optomagnetic detection, were integrated into a microfluidic chip containing three connected reaction chambers, below which a motorized permanent magnet was positioned to move analytes (captured by magnetic microparticles) along the fluid channel.

For homogeneous VAMNDAs without any washing/separation steps, the multiplex sensing can be realized by utilizing MNPs showing distinct relaxivities. As reviewed in a previous section, MNPs of different hydrodynamic volumes can be distinguished based on their characteristic χ″ peaks for multiplex detection. However, due to the influence of MNP uniformity, only biplex VAMNDAs using 100 (or 80) and 250 nm sized MNPs have been demonstrated by far (Strömberg et al., 2009; Tian et al., 2016a, 2017). Finding the third MNP size with a distinguishable magnetic signal is not easy: smaller MNPs have weaker magnetic responses, whereas larger MNPs are easier to sediment and less sensitive to size changes. In order to solve this problem, VAMNDA studies can be carried out on multi-transducer platforms that analyze signals of different energies with lock-in techniques, which requires more interdisciplinary collaborations in the future.

Portable and ease-of-use magnetic biosensing systems based on the VAMNDA concept have been commercialized for clinical applications, for example, one-drop-of-blood quantitative virus tests (blusense-diagnostics.com). However, these commercialized biosensors were realized based on the (target antibody-induced) immuno-agglutination but not NAA, which could probably be explained by the lack of reliable and sensitive homogeneous isothermal amplification strategies with proper controls of background amplification. Nevertheless, some NAA-based VAMNDAs were verified by testing clinical samples such as virus specimens (vaccine and tissue) (Tian et al., 2016b) and bacterium specimens (urine) (Mezger et al., 2015), implying potential applications in diagnosis.

## Conclusion

Following the trends of nanoscience, magnetics, and sensing techniques over the last decade, VAMNDAs have undergone a pronounced evolution toward point-of-care testing. However, despite the rapidly improved biosensing performances, technical challenges remain. We herein provide an overview of VAMNDA and introduce the concepts and performances of different NAA strategies employed in this technique. Thereafter, limitations and prospects toward point-of-care applications are discussed. We hope that this mini-review will motivate studies to help solve the current limitations that are preventing VAMNDA from clinical applications.
